# Overview of physical activity promotion in day care centres in Germany. Results of a cross-sectional survey of the BeweKi project

**DOI:** 10.25646/13519

**Published:** 2025-11-19

**Authors:** Susanne Krug, Susanne Jordan, Johanna Romefort, Loreen Neidhart, Julika Loss, Susanne Kuger

**Affiliations:** 1 Robert Koch Institute, Department of Epidemiology and Health Monitoring, Berlin, Germany; 2 German Youth Institute, Munich, Germany; 3 Heinrich Heine University, Düsseldorf, Germany; 4 Ludwig Maximilian University, Munich, Germany

**Keywords:** Day care centre, Physical activity, Promotion of physical activity, Physical activity time, Prevalence, Germany

## Abstract

**Background:**

Physical activity promotion should start early, as a physical active lifestyle is easier to establish at a young age and can have a positive impact into adulthood. Day care centres are particularly suitable for promoting physical activity, as over 90 % of children aged three and above attend a day care centre. Despite numerous programmes, there is a lack of scientific data on the prevalence of specific measures and insufficient evidence on factors influencing their implementation.

**Methods:**

The survey on the status of physical activity promotion in day care centres (BeweKi survey 2022/2023) asked day care centre directors about structural conditions and pedagogical staff about physical activity-related personal characteristics. Data is available for 1,647 day care centres.

**Results:**

Most day care centres engaged in walks, excursions, and used (external) sport halls at least once per week to promote physical activity, while digital resources or swimming pools were used very rarely. Almost all provided at least one hour of free physical activity time per day. Only a quarter offered an equally long period of structured units for physical activity, for which regular team discussions on physical activity, sufficient area in the day care centre and staff moving together with children were enabling factors.

**Conclusions:**

Physical activity promotion is widespread in day care centres, but structured physical activity time is not sufficiently established. To increase this, spatial and organisational conditions could be addressed, as well as staff physical activity behaviour.

## 1. Introduction

Regular physical activity has a preventive effect on noncommunicable diseases [1], which represent the highest disease burden in Germany [2]. Promoting physical activity from childhood onwards is one of the most effective preventive measures for reducing the risk of a wide range of physical and mental diseases [3]. Early practice of a physically active lifestyle promotes health in childhood and increases the likelihood of sufficient physical activity in adulthood [4]. International and national recommendations on promoting physical activity therefore unanimously emphasise that physical activity should be encouraged from an early age [5–9]. The World Health Organization (WHO) recommends at least 60 minutes of moderate to vigorous physical activity per day for children aged three and above [8, 9]. Since 2020, this recommendation has been considered fulfilled for children aged five and above, if a total of 420 minutes is achieved over the course of a week. Although the data from the last ten years cannot be compared due to this change in the WHO recommendation, it shows that many children in Germany do not achieve the recommended minimum amount of physical activity [10, 11]. In summary, there is both a need and potential to promote physical activity in early childhood.

**Infobox on promoting physical activity in day care centres**

**Free physical activity time**, during which children can gain physical activity experience on their own terms (e.g. with different materials), are considered effective methods for promoting physical activity in day care centres [7, 16, 17]. Since children are usually only moderately to strenuously physically active for 10 to 15 minutes during free physical activity time, it is considered more effective to have several free times throughout the day [7, 16].

**Structured units for physical activity** are led by pedagogical staff and are therefore also referred to below as **guided units**. Their aim is to ensure that all children are systematically and intensively engaged in moderate to intense physical activity for a longer period of time (e.g. through gymnastics sessions, movement stories) [7, 16, 17]. This also allows children who are less motivated or unsure about physical activity to be reached. Structured units for physical activity should take place in addition to free physical activity times and, ideally, on a daily basis.

Day care centres are particularly well suited to promoting physical activity in children, as over 90 % of children aged three and above attend day care [12]. The results of studies available to date also suggest that targeted measures in early childhood education and care settings can increase children’s physical activity [6, 7, 13, 14]. The scientific evidence for this is based on individual reviews and studies [6, 7, 13, 14]. Accordingly, the orientation, learning and education plans for day care centres in all federal states also include physical activity as a field of action [15].

Structured units and free physical activity times (Infobox) [7, 16, 17] are considered particularly effective methods for increasing children’s physical activity in daily practice in day care centres. It is also beneficial if the pedagogical staff are trained in promoting physical activity, are physically active in their free time and moving together with children in day care centre [7, 13, 14]. Structural conditions in the day care can contribute to increasing physical activity, especially the design of the indoor and outdoor areas and materials for promoting physical activity [6, 7, 13, 14, 16–18]. Other identified enabling factors are theoretically designed physical activity promotion measures [16], the implementation of comprehensive measures consisting of several interrelated components in day care practice [14], and the integration of parental involvement [16, 17]. Overall, the current state of international research shows that, on the one hand, the spatial and material conditions of the day care centre (physical activity-related structural factors) and, on the other hand, the behaviour and attitudes of the pedagogical staff (personal characteristics of the pedagogical staff) influence the physical activity of children in everyday day care life. These findings on promotion of physical activity in day care centres provide important insights into effective methods and conditions for fostering children’s physical activity in daily practice.

**Key messages**

► 95.9 % of day care centres offered at least one hour of free physical activity time per day. However, only 23.3 % implemented structured units for physical activity of the same duration each day.
► About half of the day care centres had staff with additional qualifications in the field of physical activity.
► Just over half of the day care centres had a profile with a focus on physical activity.
► Structured units for physical activity were more likely when regular team discussions on physical activity took place, when pedagogical staff were satisfied with the indoor and outdoor areas for physical activity, and when they moved together with children.
► Key approaches to strengthening structured physical activity programmes include improving spatial conditions, team discussions on physical activity, and encouraging pedagogical staff to move together with children.

In day care centres in Germany, promoting physical activity is seen as an important preventive topic [19, 20], but measures are mainly taken within the framework of individual, specific or locally limited projects [6, 20–22] such as ‘TigerKids – Successful Health Promotion in Preschool Settings’) or ‘Queb – Developing Quality with and Through Physical Activity in Childcare Centers’ [6, 23]. Little is known about the present prevalence of specific measures actually implemented in daily practice in day care centres in Germany. This is also relevant for the implementation of effective methods, such as structured (guided) units for physical activity. Preliminary information are provided by a review from 2022, for which examples of good practice in promoting physical activity in day care centres were researched in five project databases [6]. The nine projects identified reported a distribution between 63 and 5,500 day care centres throughout the project period, but the number of day care centres reached is not available for all projects [6]. The same applies to factors that currently facilitate or hinder the implementation of physical activity promotion in day care centres in Germany. There is a lack of data-based findings on the relationship between physical activity-related structural characteristics of day care centres and personal characteristics of pedagogical staff in promoting physical activity among children in day care centres. This article aims to close these two research gaps.

The **first research question** examines which measures to promote physical activity in day care centres are widespread in Germany. An overview is provided of the frequency of various measures to promote physical activity in day care centres in Germany. These include structured and free physical activity times, as well as specific physical activity offerings such as walks or the use of (external) sport halls. The **second research question** examines which physical activity-related structural characteristics of the day care centre and personal characteristics of the pedagogical staff are related to structured units for physical activity. Of interest here are those characteristics that have emerged from previous research on specific physical activity promotion and general pedagogical work as potentially important factors, such as a day care centre profile with a focus on physical activity, satisfaction of pedagogical staff with conditions in the day care centre for promoting physical activity, or pedagogical staff’s movement together with children during physical activity promotion [6, 7, 13, 24, 25].

## 2. Methods

### 2.1 Sample design and study conduct

The data from the ‘Survey on physical activity promotion in day care centres’ (BeweKi survey 2022/2023) form the basis of our analyses. The cross-sectional study surveyed day care centres in Germany about their physical activity promotion activities and the structural and personal conditions for physical activity promotion. The survey was part of the Robert Koch Institute’s (RKI) ‘Investigation of physical activity promotion in day care centres, schools and sports clubs – taking into account the pandemic’ (BeweKi).

The sample was drawn from the address database of the German Youth Institute’s (DJI) study ‘An indicator-based monitoring of structural quality in the German early childhood education and care system’ (ERiK) (as of 2020) and included only day care centres for pre-school children. Data collection was carried out from the end of 2022 to February 2023 by the infas Institute for Applied Social Science GmbH (infas). To this end, 5,500 day care centres throughout Germany were contacted in writing. The size of the gross sample was determined based on prior experience from sample size estimates from previous ERiK studies and budget restrictions. Based on these empirical values, a total response rate of approximately 26 % was expected for day care centre directors and approximately 17 % for pedagogical staff [26]. One day care centre director and one member of the pedagogical staff, selected at random based on their last birthday, were invited to take part in the survey. The questionnaire for day care centre directors covered general questions about the facility and physical activity, while the questionnaire for pedagogical staff covered questions relevant to physical activity in daily practice in the day care centre. The questions could be answered either using a printed version of the paper questionnaire (PAPI) or an online questionnaire (CAWI: Computer Assisted Web Interview). The response rate was calculated using the Outcome Rate Calculator of the American Association for Public Opinion Research (AAPOR) [27].

For the analyses, a weighting factor was created that considers different selection and participation probabilities and ensures that the prevalence estimates for certain characteristics (e.g. federal states, type of provider, day care centres size) are representative of day care centres in Germany. The responses from the questionnaires for day care centre directors and pedagogical staff were combined into a single data set for the data analysis so that reports could be made on the facility level regarding the promotion of physical activity. The weights for the analyses at the facility level were calculated in a multi-stage process involving design weighting with inverse selection probability (known as Horvitz-Thompson estimators [28]), the adjustment of the design weights (default model) and calibration based on information on the distribution of day care centres across the various federal states, the proportion of different types of providers and the size of day care centres using data from the 2022 German Child and Youth Welfare Statistics [29]. The analyses only include complete cases (n = 1,647) in which two completed questionnaires (day care centre director and pedagogical staff) are available for a day care centre. The detailed study design and measurement instruments are described in the study protocol [30].

### 2.2 Variables

#### General structural characteristics of the day care centres

The general structural information on the participating day care centres is used to describe the composition of the sample.

##### Type of provider

The day care centre director could select the type of provider from one of the following categories, which are based on key studies of day care centres in Germany [31]: public provider; nonprofit religious provider; nonprofit nonreligious provider; other nonprofit provider and, private nonprofit provider. The type of provider was dichotomised in the analyses: public versus nonstate providers (all others).

##### Day care centre size by number of children

The day care centre director was asked, ‘*How many children were being cared for in your day care on the reference date of 1 November 2022?*’ Based on the number of children, which could be entered in a free text field, the day care size was divided into three categories: ‘Small’ (≤ 25 children), ‘Medium’ (26 to ≤ 75 children) and ‘Large’ (≥ 76 children).

Information on the federal state is available for each day care centre.

#### Physical activity-related structural characteristics of day care centres

Physical activity-related structural conditions provide information about spatial, material and organisational factors that can influence the promotion of physical activity in day care centres.

##### Physical activity in the day care centre profile

The day care centre director was asked, ‘*Does your day care have an pedagogical concept or mission statement or profile with a focus on physical activity?*’ This question was to be answered with ‘Yes’ or ‘No’; other profile topics were not asked about.

##### Staff with additional qualifications in the field of physical activity

The day care centre director was asked: ‘*What percentage of pedagogical staff (including yourself ) at your day care have additional qualifications in the field of physical activity (e.g. specialist in health and physical activity promotion)?*’ The possible answers were: ‘None’, ‘1 – 10 %’, ‘11 – 20 %’, ‘21 – 30 %’, ‘31 – 40 %’, ‘More than 40 %’. The created variable ‘staff with additional qualifications’ was dichotomised into ‘No’ and ‘Yes’ and included in the regression analyses.

##### Regular team discussions on physical activity

The pedagogical staff were asked to answer the following question: ‘*The team engages in regular discussions on topics related to physical activity and possible physical activity opportunities*’. The answer should refer to the past twelve months and be given on a six-point Likert scale from ‘Strongly disagree’ to ‘Strongly agree’.

##### Own physical activity area or exercise room

The question ‘*Does your day care centre have its own room that is used exclusively as a physical activity area or exercise room?*’ could be answered by the day care centre director with ‘Yes’ or ‘No’.

##### Cooperation with external providers

The day care centre director was asked, ‘*Has your day care centre cooperated with external (regional, if applicable) sports clubs/providers in the vicinity of the day care centre in the last 12 months?*’ The question could be answered with ‘Yes’ or ‘No’ and was only asked to those day care centre directors who answered ‘Yes’ to the preceding question ‘*Are you aware of any external (regional, if applicable) sports clubs/providers in the vicinity of your day care centre?*’ Day care centre directors who answered ‘No’ to this question were assumed to have no cooperation with external sports clubs/providers.

#### Physical activity-related personal characteristics of pedagogical staff

The physical activity-related personal characteristics of the pedagogical staff describe factors at the individual level that may influence the implementation of daily physical activity promotion, such as attitudes, satisfaction, and their own physical activity-related behaviour [14].

##### Satisfaction with conditions for promoting physical activity

The pedagogical staff were asked about their satisfaction with conditions for promoting physical activity in their day care centre over the past twelve months. Response categories were on a six-point Likert scale ranging from ‘Not at all satisfied’ to ‘Very satisfied’. The analyses included: ‘Sufficient indoor area for physical activity’, ‘Sufficient outdoor area for physical activity’, ‘Indoor play and physical activity equipment (e.g. foam building blocks, mats, roller boards, balls)’, ‘Outdoor play and physical activity equipment (e.g. slide, climbing frame, swing, balance bikes, tricycles)’, ‘Own training and additional qualification opportunities’ and ‘Actual staff to child ratio’.

##### Moving together with children

Assessment of the pedagogical staff’s engagement in promoting physical activity was conducted using the question ‘*How often are you moving together with the children (e.g. in movement games, dancing)?*’ The response options were ‘Never’, ‘Rarely’, ‘Sometimes’, ‘Frequently’ and ‘Always’, which were dichotomised for the regression analyses into ‘Never/Rarely/Sometimes’ and ‘Frequently/Always’.

#### Promotion of physical activity

##### Free physical activity time

The pedagogical staff were asked: ‘*How much free physical activity time (e.g. free play involving vigorous movements such as running, romping, hopping, jumping, climbing) is available to the children on a normal day at the day care centre? Please base your answer on the majority of the children in your care*’. The response options in hours were ‘Less than 1’, ‘1 to 2’, ‘3 to 4’, ‘5 to 6’, ‘At least 7’. For the presentation of results, the categories were summarised into ‘≤ 2’, ‘3 – 4’, and ‘≥ 5’ hours.

##### Structured units for physical activity

The pedagogical staff were asked to provide information on structured units for physical activity: ‘*How much structured units for physical activity (e.g. movement songs/games, climbing courses, gymnastics lessons) do the children have in a normal week at the day care centre? Please base your answer on the majority of the children in your care. Please provide an answer for each line*’. The answer options per weekday were ‘None’, ‘*Less than one hour*’, or ‘*At least one hour each day of the week*’. For the regression analysis, the variable was dichotomised into ‘Daily’ (five days for at least one hour) and ‘Not daily’ (zero to four days for at least one hour), with the latter category representing the reference category. Data for Saturday and Sunday were not considered due to the low case numbers.

##### Specific physical activity offerings

The pedagogical staff were asked the question ‘*How often have you offered the children the following physical activity opportunities in the past 12 months?*’ in relation to ‘*Walks/excursions*’, ‘*Use of (external) sport halls/exercise rooms*’, ‘*Use of swimming pools*’, ‘*Paid physical activity activities (e.g. voluntary/additional groups for swimming, football, dance, etc.)*’ and ‘*Digital physical activity programmes (e.g. videos with physical activity examples for rhythm, dance, etc.)*’. The response options were presented on a six-point scale with ‘Daily’, ‘Several times a week’, ‘Once a week’, ‘One to three times a month’, ‘Less than once a month’ or ‘Not generally offered’.

### 2.3 Statistical methods

To answer the first research question, which measures to promote physical activity in day care centres are widespread in Germany, the prevalence is reported descriptively, stating the weighted percentages at the facility level and the 95 % confidence interval (CI).

The second research question examines whether physical activity-related structural characteristics of day care centres and personal characteristics of pedagogical staff (independent variables) are associated with achieving a structured unit for physical activity period of at least one hour per day (dependent variable). For this purpose, an unweighted binary logistic regression analysis was conducted using maximum likelihood estimation with all factors considered relevant in the literature [32] and adjusted for the general structural characteristics of the day care centres (federal state, type of provider, day care centre size). The reference category used for each variable serves as a comparison category to which the effects of the other categories on the variables to be explained are relatively estimated. The background for the unweighted regression analysis is the relatively complex study design [30] and the resulting high standard errors. At the same time, almost all weighting variables were included as control variables. The independent variables included both metric and nominal predictors. Metric, nonlinear variables were dichotomised. Cragg & Uhler’s R^2^ (also known as Nagelkerke R^2^) was calculated as a measure of the explained variance/model quality. To interpret the effect sizes, average marginal effects (AMEs) were estimated [33] to represent the influences of the predictors on the probability of at least one hour of guided unit per day. The AMEs determine the average changes in the predicted probability of at least one hour of guided unit per day when the independent variables increase by one unit. Conversely, the AMEs determine the average effect/influence of the independent variables on the probability of at least one hour of guided unit per day. The effect of each metric independent variable is presented as the change in the relative probability of at least one hour of guided physical activity unit per day for a ± one-unit change, expressed as ± (AME × 100) percentage points. The effect of nominal independent variables is described as ± (AME × 100) percentage points compared with the category chosen as the reference. In addition to the direction and strength of the association, it is considered statistically significant at a p-value ≤ 0.05. Multicollinearity among the independent variables was also assessed using the Variance Inflation Factor (VIF) test. The analyses were performed using STATA V.17 statistical software.

## 3. Results

A total of 1,647 day care centres participated in the survey on the current state of physical activity promotion. For each of these facilities, a complete interview with the day care centre director and a member of the pedagogical staff is available. The day care centres can be characterised on the basis of general structural characteristics such as type of provider, day care centre size (based on the number of children cared for) and federal state. The weighting described in 2.3 allowed the sample to be representative of day care centres in Germany according to characteristics mentioned, with reference to the Statistics on Children and Staff in Day Care Centres of the federal and state governments [34]. Annex Table 1 shows the proportion of day care centres surveyed according to these three characteristics. The results of the descriptive and regression analyses used to answer the two research questions are described below. The answer to the first research question, which measures of physical activity promotion in day care centres in Germany are implemented and to what extent, is provided by a descriptive overview of the frequency of implementation of the various measures.

### 3.1 Prevalence of physical activity promotion measures

Just over half of the day care centres (53.5 %) offered more than two hours of free physical activity time per day (Figure 1a), and three-quarters of the day care centres carried out structured units for physical activity several times a week (Figure 1b). Overall, almost all day care centres (95.9 %) provided at least one hour of free physical activity time per day, but only 23.3 % provided the same amount of time for structured units for physical activity (Figure 1c).

**Figure 2:**
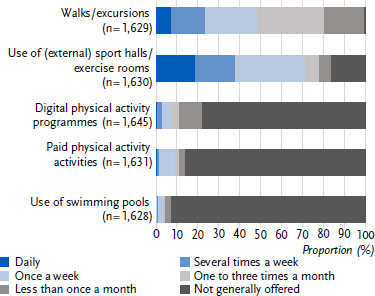
Frequency of specific physical activity offerings in the past twelve months. Percentage of day care centres (n = 1,645). Source: BeweKi survey 2022/2023

6.9 % of day care centres offered walks and excursions daily in the twelve months prior to the survey, and 41.0 % offered them once or several times a week. 18.4 % used (external) sport halls/exercise rooms to promote physical activity (Figure 2). The vast majority of day care centres did not use digital or paid exercise programmes to promote physical activity, nor did they attend swimming pools (Figure 2).

### 3.2 Frequency of structural and personal conditions for promoting physical activity in day care centres

The descriptive analyses show that 43.0 % of day care centres had a day care profile with a focus on physical activity (Table 1). Almost three quarters of day care centres (72.4 %) had their own exercise room in the twelve months prior to the survey, and 41.6 % cooperated with external providers in the day care centre’s vicinity. About half (50.9 %) had pedagogical staff with additional qualifications in the field of physical activity. 54.8 % of pedagogical staff reported that they had regular team discussions on physical activity topics and physical activity opportunities.

The high level of satisfaction among pedagogical staff with the outdoor area of their own day care centre is striking: 91.3 % were satisfied with the movement spaces (61.6 % were even very satisfied) and 85.2 % with the physical activity equipment available there (Table 2). Satisfaction with the indoor areas for physical activity was significantly lower at 57.9 %, however 74.8 % rated the indoor play and physical activity materials as satisfactory. Three-quarters (75 %) were satisfied with the opportunities for additional qualifications and training in physical activity. In contrast, only 44.8 % said they were satisfied with the actual staff-to-child ratio. Slightly more than half (55.7 %) of the pedagogical staff reported that they often moved together with children during physical activity promotion, and almost a quarter (23.5 %) said they always did so (Annex Table 2).

### 3.3 Association between structural and personal characteristics of day care centres and guided units

To answer the second research question, which physical activity-related structural characteristics of the day care centre and which personal characteristics of the pedagogical staff are associated with guided units, regression analyses were conducted. Only one structural characteristic of the day care centre is significantly associated with guided units: regular team discussions on physical activity (Table 3). With each one-point increase on the six-point scale measuring regular team discussions (‘strongly disagree’ to ‘strongly agree’), the predicted probability of guided units increases by 3.1 percentage points.

With regard to personal characteristics, the multivariate analysis shows significant associations between guided units and satisfaction with sufficient indoor and outdoor physical activity areas, as well as the pedagogical staff moving together with children (Table 3). If the pedagogical staff are satisfied with the indoor areas for physical activity, the predicted probability of structured units for physical activity (guided units) is 5.2 percentage points higher compared to those who are rather dissatisfied. If the staff are satisfied with the outdoor areas for physical activity, this increases the predicted probability of guided units by 8.7 percentage points. In addition, there is a significant association between guided units and pedagogical staff moving together with children. The predicted probability for this is 8.2 percentage points higher when the pedagogical staff frequently or always move together with children, than when they never, rarely or only sometimes move together with children. Overall, with an R^2^ of 0.115, the model explains only a small proportion of the variance.

## 4. Discussion

### 4.1 Summary of results

The prevalence of physical activity promotion in day care centres in Germany (first research question) presents a differentiated picture in the BeweKi survey 2022/2023. There were clear differences between the prevalence of free physical activity time and structured units for physical activity: almost all day care centres offered at least one hour of free physical activity time per day, but only just under a quarter provided guided units. The majority of day care centres went on walks and excursions at least once a week and used (external) sport halls/exercise rooms. Digital and paid physical activity programmes, as well as swimming pool use, played hardly any role in daily practice.

Various structural characteristics were present in a physical activity-supportive form in approximately half of the day care centres. Among the personal characteristics, staff satisfaction with the outdoor area was significantly higher compared with the indoor area. Key factors for the implementation of guided units (second research question) were found to be regular team discussions on physical activity, satisfaction with sufficient indoor and outdoor physical activity areas, and the pedagogical staff move together with children.

### 4.2 Interpretation of the results

Almost all day care centres enabled children to achieve the level of physical activity recommended by the WHO during their time at the day care centre by using the free physical activity time method. The results of BeweKi survey 2022/2023 thus confirm the findings of the ERiK study from 2020, according to which more than four-fifths of the pedagogical staff surveyed by the ERiK study considered the promotion of physical activity to be integrated into daily practice at day care centres [35]. In this way, day care centres can help to prevent or at least compensate for physical inactivity on the days when children attend the centre. This is because not all children get enough physical activity at home, but almost all of them attend day care at preschool age [12]. However, children only achieve the minimum recommendation at day care centre if they engage in moderate to strenuous physical activity for a full hour. To achieve this goal, it is better to offer several shorter periods of free physical activity time throughout the day rather than one longer period [36], as children are most active in the first 15 minutes [7]. Our study does not provide any information on the distribution of free physical activity times throughout the day. Our results on structured units for physical activity, with just under a quarter of day care centres reporting the implementation of at least one hour of structured units for physical activity per day, are clearly above the findings of the Motorik-Module study, which also recorded children’s physical activity time in day care centres between 2014 and 2017. The Motorik-Module study reported an average of 74 minutes per week of guided units for 4- to 5-year-olds [37]. However, these data are not based on information from day care centre staff, but on a physical activity questionnaire completed by the children together with their guardians [37]. The different respondents in the two studies are probably a notable reason for these differences.

In addition to free physical activity time and structured units for physical activity, walks and excursions serve as alternative forms of outdoor physical activity. They support day care centres without their own exercise rooms in promoting physical activity, which applies to approximately two-thirds of day care centres [38]. The results of BeweKi survey 2022/2023 show a higher use of outdoor forms of physical activity, such as walks and excursions, compared to data from the National Educational Panel Study (NEPS) from 2011 [39] (NEPS: 36 % vs. BeweKi survey 2022/2023: 48 %). This difference may be due to adaptation and habituation from positive experiences with these forms of outdoor physical activity during the COVID-19 pandemic [40]. The daily to at least weekly use of (external) sport halls/exercise rooms has also increased compared to NEPS from 2017 [41] (NEPS: 30 % vs. BeweKi survey 2022/2023: 41 %). This difference may be due to different question wording or the significantly smaller NEPS sample and points to a need for further research. In making this comparison, it should also be taken into account that in recent years various initiatives have addressed awareness of physical activity in public institutions and in the general population [6, 42, 43].

Digital and parent-funded physical activity programmes, as well as the use of swimming pools, were rarely part of the daily practice in the day care centres. Compared to NEPS data from 2017, swimming pools were used only half as often by day care centres in the BeweKi survey 2022/2023 [41] (NEPS: 15 % vs. BeweKi survey 2022/2023: 7.1 %). The preventive potential of early water familiarisation and preparation for learning to swim is offset by costs and a recommended staff-to-child ratio of 1 : 5 [44]. In fact, in 2022, one pedagogical staff member was responsible for an average of 7.8 children aged three and above until they started school (depending on the federal state, the figure ranges from 6.5 to 11.9) [45]. The available working time is a decisive criterion not only for specific physical activity offerings, but also for the development of a certified physical activity focus or the qualification of staff in the field of physical activity [46]. In general, it can be assumed that a better staff-to-child ratio contributes to increasing the number of structured units for physical activity in day care centres. In this respect, the second law on the further development of quality and participation in the early childhood education and care settings (Day Care Centre Quality Act) is to be welcomed, as it names ‘promoting child development, health, nutrition and physical activity’ as one of seven areas of action [47]. Financial support from the federal states of around eight billion euros from 2023 to 2026 is intended, among other things, to help strengthen the staff-to-child ratio and promote physical activity, although the promotion of physical activity can mean different things in each federal state depending on the orientation, learning and education plans [15].

Depending on the characteristic, approximately half to two-thirds of the day care centres reported physical activity-related structural conditions in their day care centres that are known from research to increase physical activity promotion in day care centre practice [48]. These included a day care centre profile with a focus on physical activity, pedagogical staff with additional qualifications in the field of physical activity, regular team discussions on physical activity topics and possible physical activity offerings, cooperation with external providers, and the availability of a dedicated physical activity or exercise room. The relatively high prevalence confirms the increased awareness of the importance of physical activity in the daily pedagogical routine of day care centres in politics and practice. The German Sports Youth (DSJ) advocates for implementation and financing options for implementing quality standards in day care centres and for providing advice on these standards [49]. In Germany, as part of the ‘Physical Activity and Health’ round table, which was coordinated by the Federal Ministry of Health from 2022 to 2023, it was agreed, among other things, to strengthen physical activity in day care centres as a measure to improve the promotion of physical activity [43], and in the Day Care Centre Quality Act, the promotion of physical activity is part of an addressed field of action [47].

Attitudes and behaviours of pedagogical staff are key to the amount of children’s physical activity levels at day care centres [22] and therefore underscore the importance of staff’s physical activity-related personal characteristics. In BeweKi survey 2022/2023, the high level of satisfaction among pedagogical staff with the outdoor area and equipment was striking, especially when compared to their satisfaction with the available indoor area. The difference was not as great for indoor play and physical activity equipment. The high level of satisfaction with the outdoor area reflects the findings of NEPS in 2011, according to which 98 % of day care centres had an outdoor area of at least 100 square metres and 84 % of day care centre directors were satisfied with the outdoor area of their day care centre [39]. The nationwide 2024 Day Care Centre Report by the ‘Paritätischer Gesamtverband’ (a German national association of nonprofit organisations) also shows that 45 % of day care centres consider the indoor area to be insufficient to meet the children’s need for physical activity, but only 22 % were dissatisfied with the outdoor area [24]. In addition to the association between satisfaction with spatial and organisational conditions for physical activity, the results also show an association between physical activity behaviour of pedagogical staff and the promotion of physical activity in day care centres. Various studies have shown that active participation by pedagogical staff not only motivates children more and increases their range of physical activity, but also increases the amount of physical activity undertaken by pedagogical staff, with positive effects on their health [22, 50, 51]. However, as moving together with children during physical activity promotion is not part of the daily practice in day care centres – as shown by data from BeweKi survey 2022/2023 – this area should be further developed in additional qualification and training. In this way, children can be encouraged to enjoy physical activity and be presented with a model of active adulthood.

The implementation of at least one hour of structured units for physical activity per day is more likely when there are regular internal team discussions on physical activity. This can be seen as an indicator of structured discussion within the team and of quality assurance in the promotion of physical activity. However, the existence of a day care centre profile in the area of physical activity does not increase the likelihood of structured units for physical activity, contrary to what the state of research would suggest. One reason for this could be that labels or certifications for day care centres involve a great deal of time-consuming documentation and tie up resources [46]. Another explanation could be that physical activity and nutrition are often included together in the day care centre profile and are therefore not necessarily accompanied by active engagement in how structured units for physical activity can be implemented in daily practice. The existence of a day care centre profile in the area of physical activity may also be the result of training courses that took place some time ago. The lack of association could also be due to the time frame of the question, which referred to the last twelve months. Participation in additional training and qualification may still have been affected by contact restrictions and the additional burdens of the COVID-19 pandemic. A recent study showed that day care centre directors were not familiar with the details of physical activity recommendations despite the day care centre’s physical activity label [46] and were therefore perhaps also unaware of the effects of regular structured units for physical activity. The importance of pedagogical staff moving together with children was also a strongly fostering factor in the overall analysis and confirms the existing evidence [22, 50, 51]. The implementation of structured units for physical activity was more likely if the pedagogical staff rated the indoor and outdoor areas as adequate. Equipment or an unfavourable staff-to-child ratio, on the other hand, seemed to be less important. The finding that attitudes towards physical activity and the promotion of physical activity are more important for structured units for physical activity than organisational conditions should be investigated in more depth in further research.

### 4.3 Limitations and strengths

A major strength of the study is the large nationwide sample, which allows for representative results [30]. Including the day care centre director and the pedagogical staff in each day care centre provides insights into structural and personal factors, as well as varied information on the daily practice of physical activity promotion, from two perspectives. This is a methodological approach that is also used in other large studies [52]. Another strength lies in the simultaneous examination of structural and personal conditions for physical activity promotion in day care centres. Despite its strengths, the study has some limitations. The cross-sectional design provides data for a specific survey period; therefore, only statistical associations between various factors and indicators of physical activity promotion in day care centres can be examined, and causal relationships cannot be inferred [53]. The inclusion of a maximum of two people from each day care centre may not fully reflect the extent and diversity of physical activity promotion carried out in the day care centre, especially in larger day care centres. The practice of promoting physical activity among children can vary from group to group and from staff member to staff member, even within a single day care centre. As the data is self-reported by day care centre directors and pedagogical staff, there may be bias due to memory errors or socially desirable responses, e.g. interest-driven responses from the respective professional groups [54]. In addition, some of the questions in the questionnaire were developed in-house and have not been fully validated. The survey only contains data based on assessments by day care centre directors or pedagogical staff. The opinions of parents or even the children themselves would supplement this information with further insights, as would objective measurements of the children’s physical activity. However, the aim of our study was to gain insights into daily physical activity promotion practices and not to record the actual physical activity behaviour of the children. It is therefore not possible to assess how different measures or structures in a day care centre affect the actual physical activity behaviour of the children. Other study designs are required to evaluate the effectiveness of various measures to promote physical activity.

### 4.4 Conclusions

Since many children do not meet the WHO minimum recommendations for physical activity during their leisure time and at home, and most children attend early childhood education settings, day care centres are particularly well suited to make a substantial contribution to helping children meeting the recommended levels of physical activity. Promotion of physical activity in day care centres is highly important for health equity, as nearly all children, regardless of their social background, can be reached there. Even though promoting physical activity is part of daily practices in many day care centres, the majority of day care centres should increase the amount of structured units for physical activity. For pedagogical reasons, this should not be at the expense of free physical activity time. To strengthen physical activity promotion, both the satisfaction and attitudes of pedagogical staff, as well as structural and spatial-organisational factors, need to be addressed. Approaches that can support this are theory-based and include multiple components [14], consider different quality areas [49], make day care centres a healthy environment for all involved and also address parents [22, 55]. It is well known that employees in day care centre also often experience health-related strain at work [56] and would benefit from health-promoting measures of a good healthy day care centre that aims at comprehensive health and organisational development while integrating physical activity promotion [57, 58].

## Figures and Tables

**Figure 1a:**
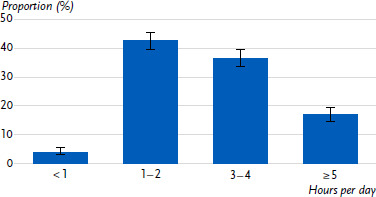
Free physical activity time in day care centres by hours per day. Proportion of day care centres in percent (n = 1,645). Source: BeweKi survey 2022/2023

**Figure 1b:**
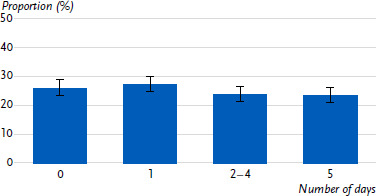
Structured units for physical activity in day care centres by days per week with at least one hour. Proportion of day care centres in percent (n = 1,611). Source: BeweKi survey 2022/2023

**Figure 1c:**
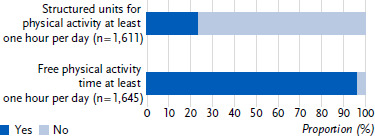
Structured units for physical activity and free physical activity time at least one hour per day (n = 1,645), respectively. Proportion in percent. Source: BeweKi survey 2022/2023

**Table 1:** Physical activity-related structural characteristics in day care centres. Proportion in percent (n = 1,647). Source: BeweKi survey 2022/2023

Physical activity-related structural characteristics	%^*^	(95 % CI)^*^
**Physical activity in the day care centre profile** (n = 1,639)		
No	57.0	(53.7 – 60.2)
Ye s	43.0	(39.8 – 46.3)
**Own physical activity area or exercise room** (n = 1,646)		
No	27.6	(24.7 – 30.7)
Yes	72.4	(69.3 – 75.3)
**Cooperation with external providers** (n = 1,640)		
No	58.4	(55.2 – 61.5)
Yes	41.6	(38.5 – 44.8)
**Staff with additional qualifications in the field of physical activity** (n = 1,645)		
None	49.1	(45.9 – 52.3)
1 – 10 %	32.7	(29.8 – 35.8)
11 – 20 %	7.8	(6.2 – 9.8)
21 – 30 %	2.3	(1.6 – 3.3)
31 – 40 %	1.9	(1.1 – 3.0)
More than 40 %	6.2	(4.8 – 8.1)
**Regular team discussions on physical activity** (n = 1,644)		
1 - Strongly disagree	8.4	(6.7 – 10.4)
2	16.4	(14.2 – 18.8)
3	20.3	(17.9 – 23.0)
4	23.3	(20.7 – 26.2)
5	19.3	(16.9 – 22.1)
6 - Strongly agree	12.2	(10.2 – 14.7)

95 % CI = 95 % confidence interval

^*^weighted results

**Table 2:** Physical activity-related personal characteristics of pedagogical staff in day care centres: satisfaction with conditions for promoting physical activity. Percentage of day care centres^*^ (n = 1,647). Source: BeweKi survey 2022/2023

Satisfaction with	1 Not at all satisfied	2	3	4	5	6 Very satisfied
	% (95 % CI)	% (95 % CI)	% (95 % CI)	% (95 % CI)	% (95 % CI)	% (95 % CI)
**Indoor area for physical activity**s (n = 1,634)	8.2 (6.7 – 10.0)	15.2 (13.0 – 17.7)	18.7 (16.3 – 21.4)	16.8 (14.5 – 19.4)	23.5 (20.9 – 26.5)	17.6 (15.3 – 20.1)
**Outdoor area for physical activity** (n = 1,638)	1.4 (0.8 – 2.4)	2.1 (1.5 – 3.1)	5.2 (4.0 – 6.8)	8.4 (6.7 – 10.5)	21.3 (18.8 – 24.0)	61.6 (58.4 – 64.7)
**Indoor play and physical activity equipment** (n = 1,636)	2.4 (1.7 – 3.5)	8.1 (6.5 – 10.1)	14.8 (12.5 – 17.3)	18.8 (16.4 – 21.4)	28.0 (25.1 – 31.0)	28.0 (25.2 – 30.9)
**Outdoor play and physical activity equipment** (n = 1,630)	1.4 (0.9 – 2.4)	3.7 (2.7 – 5.1)	9.6 (7.8 – 11.6)	12.5 (10.5 – 14.8)	27.5 (24.7 – 30.5)	45.2 (42.0 – 48.5)
**Own training and additional qualification opportunities** (n = 1,634)	3.7 (2.7 – 5.0)	7.0 (5.5 – 8.7)	14.3 (12.3 – 16.6)	17.7 (15.4 – 20.3)	32.2 (29.2 – 35.3)	25.1 (22.4 – 28.1)
**Actual staff-to-child ratio** (n = 1,635)	14.5 (12.5 – 16.7)	19.7 (17.3 – 22.3)	21.0 (18.6 – 23.7)	16.0 (13.8 – 18.5)	17.2 (14.9 – 19.9)	11.6 (9.3 – 14.2)

95 % CI = 95% confidence interval

^*^weighted results

**Table 3:** Predicted changes in the probability of structured physical activity time of at least one hour per day (reference group ‘not daily’) depending on structural and personal characteristics of the day care centre. Average marginal effects (AME in percentage points) of the binary logistic regression model (n = 1,516; complete cases). Source: BeweKi survey 2022/2023

Predictors (independent variables)		AME	(95 % CI)	p-value
**Physical activity-related structural characteristics of day care centres**				
Physical activity in the day care profile	No (Ref.)			
	Yes	3.7	(- 0.71 – 8.21)	0.099
Staff with additional qualifications in the field of physical activity	No (Ref.)			
	Yes	1.1	(- 3.36 – 5.48)	0.639
**Regular team discussion on physical activity**	‘Strongly disagree’ to ‘Strongly agree’ (six-point Likert scale)	**3.1**	**(1.50 – 4.63)**	**0.000**
Own physical activity area or exercise room	No (Ref.)			
	Yes	2.3	(- 2.98 – 7.58)	0.393
Cooperation with external providers	No (Ref.)			
	Yes	- 0.5	(- 4.88 – 3.97)	0.841
**Physical activity-related personal characteristics of pedagogical staff**				
**Satisfaction with sufficient indoor area for physical activity**	Rather no (Ref.)			
	Rather yes	**5.2**	**(0.22 – 10.14)**	**0.041**
**Satisfaction with sufficient outdoor area for physical activity**	Rather no (Ref.)			
	Rather yes	**8.7**	**(1.45 – 15.89)**	**0.019**
Satisfaction with availability of indoor play and physical activity equipment	Rather no (Ref.)			
	Rather yes	- 1.7	(- 7.56 – 4.20)	0.575
Satisfaction with the availability of outdoor play and physical activity equipment	Rather no (Ref.)			
	Rather yes	- 1.6	(- 8.53 – 5.39)	0.658
Satisfaction with own training and additional qualification opportunities	‘Not at all satisfied’ to ‘Very satisfied’ (six-point Likert scale)	- 0.2	(- 1.84 – 1.50)	0.838
Satisfaction with actual staff-to-child ratio	‘Not at all satisfied’ to ‘Very satisfied’ (six-point Likert scale)	0.7	(- 0.77 – 2.18)	0.349
**Moving together with children**	Never/Rarely/Some-times (Ref.)			
	Frequently/Always	**8.2**	**(2.48 – 13.95)**	**0.005**
Cragg & Uhler‘s R^2^/Nagelkerke R^2^ = 0.115				

AME = average marginal effect, 95 % CI = 95 % confidence interval, Ref. = reference group

**Annex Table 1:** Sample description based on the general structural characteristics of day care centres (n = 1,647). Source: BeweKi survey 2022/2023

Characteristics of day care centres	%^*^	(95 % CI)	n**
**Federal state** (n = 1,647)			
Baden-Württemberg	17.2	(14.6 – 20.2)	137
Bavaria	14.5	(12.3 – 17.1)	170
Berlin	5.2	(3.9 – 7.0)	79
Brandenburg	3.1	(2.5 – 3.8)	95
Bremen	0.9	(0.5 – 1.4)	34
Hamburg	2.2	(1.5 – 3.2)	40
Hesse	8.0	(6.5 – 9.9)	132
Mecklenburg-Vorpommern	1.8	(1.4 – 2.3)	81
Lower Saxony	9.9	(8.1 – 12.0)	139
North Rhine-Westphalia	18.2	(15.6 – 21.2)	155
Rhineland-Palatinate	4.8	(3.8 – 5.9)	122
Saarland	1.0	(0.7 – 1.3)	50
Saxony	4.6	(3.7 – 5.7)	122
Saxony-Anhalt	2.8	(2.2 – 3.5)	87
Schleswig-Holstein	3.4	(2.6 – 4.4)	105
Thuringia	2.5	(2.0 – 3.2)	99
**Type of institution** (n = 1,626)			
Nonstate provider	67.8	(64.8 – 70.6)	1,048
Public	32.2	(29.4 – 35.2)	578
**Day care size by number of children** (n = 1,633)			
Small (up to 25 children)	16.9	(14.1 – 20.1)	138
Medium (26 to 75 children)	54.6	(51.4 – 57.9)	823
Large (76 children and above)	28.5	(26.0 – 31.2)	672

95 % CI = 95 % confidence interval

^*^weighted

^**^unweighted

**Annex Table 2:** Physical activity-related personal characteristics of pedagogical staff in day care centres: Pedagogical staff moving together with children (n = 1,639). Source: BeweKi survey 2022/2023

Moving together with children	%	(95 % CI)
Never	0.2	(0.0 – 1.1)
Rarely	2.0	(1.3 – 3.1)
Sometimes	18.6	(16.3 – 21.2)
Frequently	55.7	(52.5 – 58.9)
Always	23.5	(20.8 – 26.3)

95 % CI = 95 % confidence interval
